# Dissecting the role of the ϕ29 terminal protein DNA binding residues in viral DNA replication

**DOI:** 10.1093/nar/gkv127

**Published:** 2015-02-26

**Authors:** Isabel Holguera, Daniel Muñoz-Espín, Margarita Salas

**Affiliations:** Instituto de Biología Molecular ‘Eladio Viñuela’ (Consejo Superior de Investigaciones Científicas), Centro de Biología Molecular ‘Severo Ochoa’ (Consejo Superior de Investigaciones Científicas–Universidad Autónoma de Madrid), Universidad Autónoma, Cantoblanco, 28049 Madrid, Spain

## Abstract

Phage ϕ29 DNA replication takes place by a protein-priming mechanism in which the viral DNA polymerase catalyses the covalent linkage of the initiating nucleotide to a specific serine residue of the terminal protein (TP). The N-terminal domain of the ϕ29 TP has been shown to bind to the host DNA in a sequence-independent manner and this binding is essential for the TP nucleoid localisation and for an efficient viral DNA replication *in vivo*. In the present work we have studied the involvement of the TP N-terminal domain residues responsible for DNA binding in the different stages of viral DNA replication by assaying the *in vitro* activity of purified TP N-terminal mutant proteins. The results show that mutation of TP residues involved in DNA binding affects the catalytic activity of the DNA polymerase in initiation, as the K_m_ for the initiating nucleotide is increased when these mutant proteins are used as primers. Importantly, this initiation defect was relieved by using the ϕ29 double-stranded DNA binding protein p6 in the reaction, which decreased the K_m_ of the DNA polymerase for dATP about 130–190 fold. Furthermore, the TP N-terminal domain was shown to be required both for a proper interaction with the DNA polymerase and for an efficient viral DNA amplification.

## INTRODUCTION

Replication of linear genomes poses the problem of duplicating the ends of DNA without losing information at the lagging strand. To solve this problem, different mechanisms have evolved. Phages such as T4, T7 and SPP1 produce head–tail concatemers by using redundancies at the end of their linear genomes that regenerate terminal sequences by further replication and recombination. In the case of phage λ, it circularises its linear genome and duplicates it by rolling-circle replication (reviewed in 1). Other organisms, such as prokaryotic (ϕ29, PRD1 and Cp-1) and eukaryotic (adenovirus) viruses, use a protein as primer of the replication process (2,3). In this mechanism, a serine, threonine or tyrosine residue of a specific protein called terminal protein (TP) provides the hydroxyl group needed to initiate replication.

**Figure 1.**
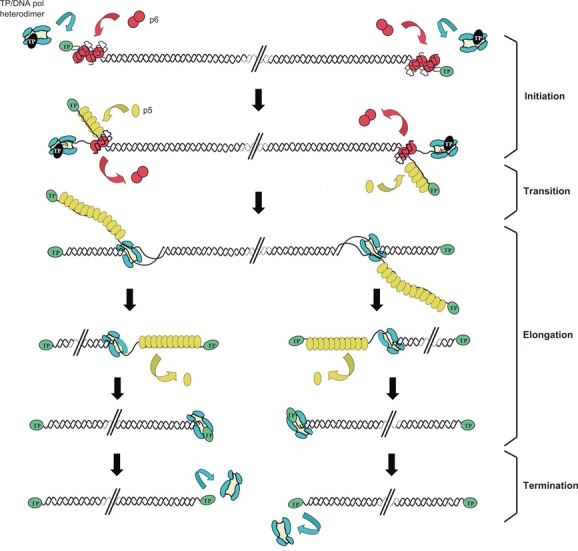
Schematic representation of the mechanism of ϕ29 DNA replication *in vitro*. The primer TP/DNA polymerase heterodimer recognises the p6-complexed replication origins. The DNA polymerase then catalyses the covalent linkage between dAMP and the hydroxyl group of TP Ser232. After a transition step (not drawn in the figure), the DNA polymerase dissociates from the TP and continues processive elongation coupled to strand displacement. Viral protein SSB p5 binds to the ssDNA-displaced strands and is further removed during the polymerisation process. Continuous elongation by the DNA polymerase from both ends leads to the complete duplication of parental strands. Green ovals: parental TP; black ovals: primer TP; red circles: p6; blue: DNA polymerase; yellow ovals: SSB p5. Linear dsDNA is shown as a double helix.

The protein-priming mechanism of DNA replication has been extensively studied using the *Bacillus subtilis* bacteriophage ϕ29 (see Figure 1 for an schematic overview of the ϕ29 replication mechanism *in vitro*). The ϕ29 genome is a linear dsDNA 19285 bp-long with a protein covalently linked to the 5′ ends (parental TP). The origins of replication consist of 12 bp terminal sequences containing inverted terminal repeats (ITRs) of 6 bp and the TP linked to the terminal nucleotide (4). To initiate DNA replication, a free TP molecule (primer TP) and the viral DNA polymerase form a heterodimer that recognises the origins of replication. A serine residue of the TP (Ser232) supplies the hydroxyl group for the formation of the TP-dAMP initiation complex in a reaction catalysed by the phage DNA polymerase (5,6), the primer TP remaining covalently bound to the 5′ ends of the viral genome. The initiation reaction is directed by the second nucleotide at the 3′ ends of the template (3′ TTT…5′), and the TP-dAMP initiation complex slides back to recover the terminal nucleotide prior to elongation (7). There is a transition stage between TP-primed initiation and DNA-primed elongation, which involves the dissociation of the DNA polymerase from the TP after the tenth nucleotide has been incorporated (8,9). Then, the DNA polymerase continues processive elongation coupled to strand displacement until the complete duplication of the parental strands.

The viral protein p6, which binds dsDNA, stimulates both the initiation and the transition steps of ϕ29 DNA replication *in vitro* (10–12) through the formation of a nucleoprotein complex at the replication origins (13–15). Although being essential for *in vivo* ϕ29 DNA replication (16–17), there is not an absolute requirement of p6 in the *in vitro* replication system. Indeed, it has been shown that the only proteins necessary to duplicate template TP-DNA *in vitro* are the viral DNA polymerase and the TP (18). However, both proteins, as well as p6 and the phage single-stranded DNA binding protein p5 (SSB) are needed to amplify TP-DNA *in vitro* (19).

The resolution of the crystallographic structure of the ϕ29 TP/DNA polymerase heterodimer has shown that the TP has an elongated three-domain structure comprising an N-terminal domain (residues 1–73), an intermediate domain (residues 74–172) that confers specificity to the interaction with the DNA polymerase (20), and a C-terminal domain (residues 173–266) that contains the priming residue (9). Both intermediate and C-terminal domains make extensive contacts with the DNA polymerase (9), and residues from these domains important for *in vitro* viral DNA replication have been described (21–25). The structure of the N-terminal domain of the ϕ29 TP could not be determined as it was disordered (9). Nevertheless, secondary structure predictions showed that this domain is composed of two α-helices connected by a disordered loop, and circular dichroism spectroscopy analyses showed that it has a substantial content (about 60%) of α-helix (26).

Previous work has shown that ϕ29 TP possesses DNA binding capacity both *in vitro* (27,28) and *in vivo* (26). The TP domain endowed with this capacity was shown to be the N-terminal domain (28), which has a basic character, being 23% of its residues positively charged. By site-directed mutagenesis it was shown that mutant TPs in the N-terminal residues K27 and K25/K27 were highly affected in DNA binding, and mutants in residues R19 and K32/K33/K34 were moderately affected, whereas mutation of residue K25 had a wild-type phenotype. On the other hand, deletion mutant TP ΔNt, lacking the N-terminal domain, was severely affected in DNA binding (26,28). The TP N-terminal domain was shown to be necessary to localise the TP and, consequently, the DNA polymerase, to the bacterial nucleoid (26), and this process is essential for an efficient viral DNA replication *in vivo* (26,28). To gain insight into the contribution of the TP DNA binding function in viral DNA replication, we studied the effect of TP N-terminal domain mutations in the different stages of ϕ29 DNA replication *in vitro*, namely initiation, transition, replication and amplification. We show that the TP N-terminal mutants deficient in DNA binding are also impaired in TP-DNA replication *in vitro*. The viral protein p6 relieves the ϕ29 DNA replication impairment by decreasing the K_m_ value of the DNA polymerase for the initiating nucleotide. In addition, the TP N-terminal domain was shown to be required both for a proper interaction with the DNA polymerase and for an efficient viral DNA amplification.

## MATERIALS AND METHODS

### Nucleotides and DNAs

Unlabelled nucleotides were supplied by Amersham Pharmacia and [α-^32^P]dATP (3000 Ci/mmol) was supplied by Perkin Elmer Inc. ϕ29 TP-DNA was obtained as described (29).

### Proteins

Strep-tagged wild-type and N-terminal mutant TPs were purified as previously described (26). TP mutant S232C was purified as described (21). Wild-type ϕ29 DNA polymerase and the exonuclease deficient mutants N62D (30) and D12A/D66A (31) were purified essentially as described (32). ϕ29 double-stranded DNA binding protein p6 and ϕ29 single-stranded DNA binding protein p5 (SSB) were purified as described (33,34).

### Protein-primed initiation of ϕ29 TP-DNA replication

The reaction mixture contained, in 25 μl, 50 mM Tris-HCl, pH 7.5, 0.1 mg/ml BSA, 20 mM ammonium sulphate, 1 mM dithiothreitol (DTT), 4% (v/v) glycerol and 0.2 μM dATP (1 μCi [α-^32^P]dATP). To analyse the template-directed TP-dAMP formation capacity of the wild-type and N-terminal mutant TPs, 1.6 nM of ϕ29 TP-DNA was used as template, and 12 nM of either wild-type or mutant TPs were incubated with 12 nM of DNA polymerase for 2 min at 30°C either in the absence or in the presence of 35 μM of ϕ29 protein p6 using 10 mM MgCl_2_ as metal activator. In the case of the template-independent TP-deoxynucleotidylation reaction, 24 nM of either wild-type or mutant TPs were incubated with 24 nM of DNA polymerase for 1 h at 30°C in the presence of 1 mM MnCl_2_. The reactions were stopped by adding 10 mM EDTA-0.1% SDS final concentration and filtered through Sephadex G-50 spin columns. Samples were analysed in SDS-12% PAGE and the TP-dAMP complex was quantified by densitometric scanning of the autoradiographs with Quantity One^®^ software. In all the reactions, the indicated amounts of either wild-type or mutant TPs were pre-incubated with DNA polymerase for 30 min at 4°C.

### Interference assay for DNA polymerase binding

To analyse the capacity of the N-terminal mutant TPs to compete with the wild-type TP for the binding with the DNA polymerase, the experiment was carried out in the same conditions as in the template-independent TP-dAMP formation but adding increasing amounts of the mutant TP: 0, 24, 48, 96 and 192 nM, to a fixed amount (24 nM) of either wild-type TP or S232C mutant TP (as control for the interference assay) in the presence of 24 nM of DNA polymerase. To analyse the competition of the TP mutant ΔNt either in the absence or in the presence (35 μM) of protein p6, the experiment was carried out in the same conditions as in the template-directed initiation reaction but adding increasing amounts of the mutant TP: 0, 12, 24, 48 and 96 nM, to a fixed amount (12 nM) of either wild-type TP or S232C mutant TP (as control for the interference assay) in the presence of 12 nM of DNA polymerase. After incubation, reactions were stopped and analysed as indicated in the protein-primed initiation section. Labelled bands were quantified by densitometry and the value obtained competing the TP S232C with each mutant TP was subtracted to the value obtained competing the wild-type TP with the corresponding mutant TP. As a control, wild-type TP was competed with increasing amounts of S232C mutant protein.

### Measurement of nucleotide binding affinity

Analysis of the Michaelis constant (K_m_) for nucleotide binding in the TP-DNA initiation reaction was carried out using 480 nM of either wild-type or mutant TP and 12 nM of the exonuclease deficient DNA polymerase D12A/D66A (31). Increasing concentrations of dATP (3 μCi [α-^32^P]dATP) ranging from 0.5 to 2000 μM in the absence of protein p6 or ranging from 0.125 to 100 μM in the presence of 35 μM of protein p6 were used. Incubation was for 4 min at 30°C using 10 mM MgCl_2_ as metal activator and 1.6 nM TP-DNA as template. Quantification was done by densitometric scanning of the autoradiographs with Quantity One^®^ software. Apparent values for K_m_ were obtained by least squares nonlinear regression to a rectangular hyperbola using Kaleidagraph 3.6.4 software.

### Transition assay

The reaction mixture contained, in 25 μl, 50 mM Tris-HCl, pH 7.5, 0.1 mg/ml BSA, 20 mM ammonium sulphate, 1 mM DTT, 4% (v/v) glycerol, 5 μM each dATP (1 μCi [α-^32^P]dATP), dGTP and dTTP (missing dCTP), 12 nM of either wild-type or mutant TP, 12 nM of DNA polymerase mutant N62D, which has impaired 3′-5′ exonuclease activity but retains wild-type strand-displacement capacity (30), and 1.6 nM of TP-DNA. Incubation was for 5 min at 30°C either in the absence or in the presence of 35 μM of ϕ29 protein p6 using 10 mM MgCl_2_ as metal activator. The reactions were stopped by adding 10 mM EDTA-0.1% SDS final concentration and filtered through Sephadex G-50 spin columns. Samples were analysed in high-resolution SDS-12% polyacrylamide gels (360 × 280 × 0.5 mm) to distinguish the TP bound to the first elongation products. In all reactions the indicated amounts of either wild-type or mutant TPs were pre-incubated with DNA polymerase for 30 min at 4°C.

### ϕ29 TP-DNA replication

The incubation mixture contained, in 25 μl, 50 mM Tris-HCl, pH 7.5, 0.1 mg/ml BSA, 20 mM ammonium sulphate, 1 mM DTT, 4% (v/v) glycerol, 20 μM each dNTP (1 μCi [α-^32^P]dATP), 12 nM of either wild-type or mutant TP and 12 nM of DNA polymerase. Reactions were carried out for the indicated times at 30°C either in the absence or in the presence of 35 μM of ϕ29 protein p6 using 10 mM MgCl_2_ as metal activator and 1.6 nM of ϕ29 TP-DNA as template. Reactions were stopped with 10 mM EDTA-0.1% SDS final concentration and filtered through Sephadex G-50 spin columns. Relative activity was calculated from the Cerenkov radiation corresponding to the excluded volume. The labelled DNA was denatured by treatment with 0.7 M NaOH, and subjected to electrophoresis in alkaline 0.7% agarose gels, as described (35). After electrophoresis, gels were dried and autoradiographed. In all reactions, the indicated amounts of either wild-type or mutant TPs were pre-incubated with DNA polymerase for 30 min at 4°C.

### ϕ29 TP-DNA amplification

The reaction mixture contained, in 25 μl, 50 mM Tris-HCl, pH 7.5, 0.1 mg/ml BSA, 20 mM ammonium sulphate, 1 mM DTT, 4% (v/v) glycerol, 80 μM each dNTP (1 μCi [α-^32^P]dATP), 10 mM MgCl_2_, 35 μM of ϕ29 protein p6, 30 μM of ϕ29 SSB, 16 pM of ϕ29 TP-DNA, 6 nM of either wild-type or mutant TP and 6 nM of DNA polymerase. Either wild-type or mutant TPs were pre-incubated with DNA polymerase for 30 min at 4°C. After incubation at 30°C for the indicated times, samples were processed as described for the TP-DNA replication assay.

To determine the functionality of mutant TP ΔNt as parental TP, reactions were carried out for 90 min at 30°C under the same conditions described above but starting with 600 μl of reaction. After 90 min of incubation, samples were splitted in three, one was supplemented with the wild-type TP/DNA polymerase heterodimer, another one was supplemented with the TP ΔNt/DNA polymerase heterodimer and the other was supplemented with the same volume of the protein dilution buffer (25 mM Tris-HCl, pH 7.5, 100 mM NaCl, 0.05% Tween20). Reactions were allowed to proceed for additional 150 min, samples were withdrawn at the indicated times and then processed as described for the TP-DNA replication assay.

## RESULTS

### TP N-terminal mutants affected in DNA binding are impaired in protein-primed initiation of ϕ29 TP-DNA replication

**Figure 2.**
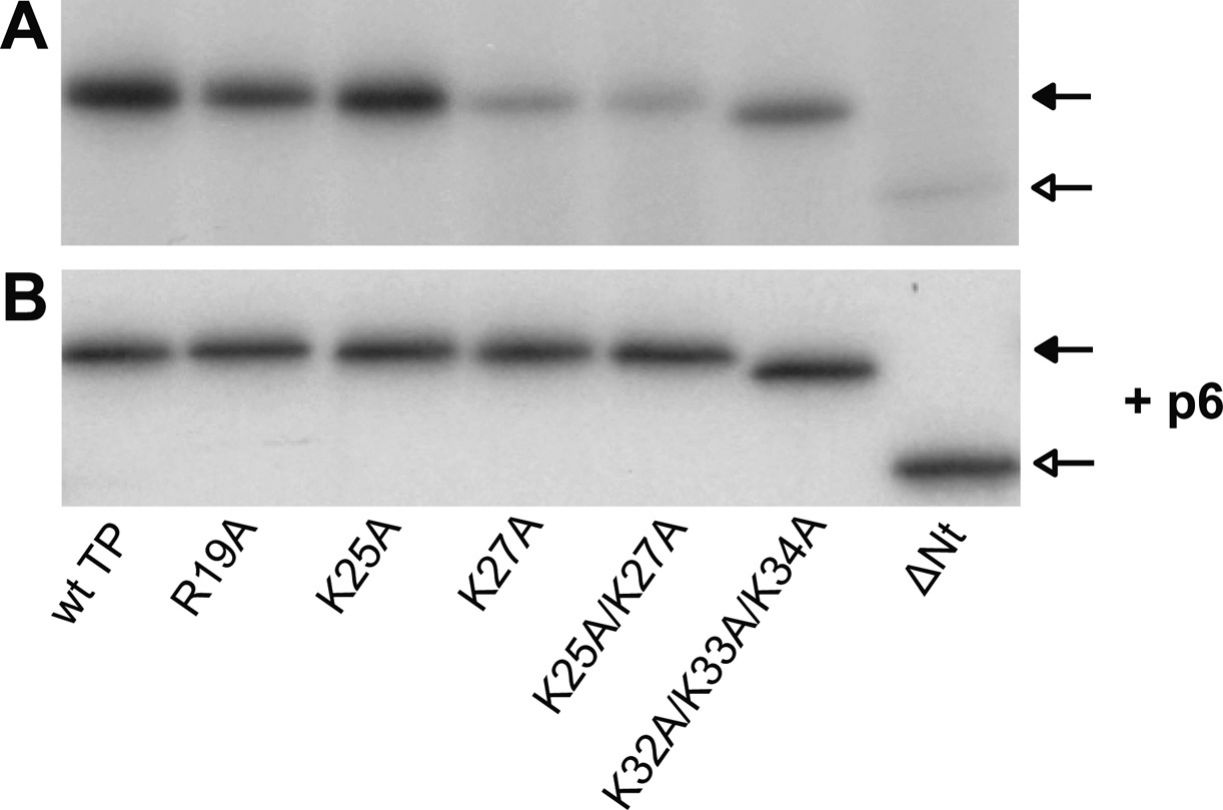
Protein-primed initiation of ϕ29 TP-DNA replication with wild-type or N-terminal mutant TPs either in the absence (**A**) or in the presence (**B**) of protein p6. The initiation assays were performed as described in Materials and Methods in the presence of 12 nM of TP, 12 nM of DNA polymerase and 1.6 nM of ϕ29 TP-DNA as template either in the absence or in the presence of 35 μM of protein p6. After incubation for 2 min at 30°C, samples were processed as described in Materials and Methods, analysed by SDS-PAGE and quantified by densitometric analysis of the autoradiographs. The figure is representative of three independent experiments. Closed arrows indicate the position of TP-dAMP and open arrows indicate the position of ΔNt TP-dAMP.

To determine if the TP N-terminal domain residues R19, K25 and K27, and the combination of residues K25/K27 and K32/K33/K34 are involved in the initial stage of the replication process, we changed each amino acid to alanine and purified the resulting proteins, as well as the TP ΔNt deletion mutant lacking the N-terminal domain (residues 1–73) (26). We analysed the formation of the TP-dAMP initiation complex by these mutant TPs using ϕ29 TP-DNA as template. Figure 2A and Table 1 show that TP mutant proteins R19A and K25A displayed essentially wild-type activity and TP mutant K32A/K33A/K34A was moderately affected in the formation of the TP-dAMP initiation complex. However, TP mutants K27A, K25A/K27A and ΔNt were highly affected in the initiation reaction. Increasing amounts of these mutant TPs (up to 40-fold that of the wild-type TP) did not change the relative initiation activity of the mutant TPs compared to that of the wild-type TP (Supplementary Figure S1).

**Table 1.** Activity of wild-type and N-terminal mutant TPs

	TP-dAMP formation^a^			TP-DNA replication^a^		TP-DNA amplification	
	TP-DNA		No template				
TP	− p6	+ p6		− p6	+ p6	%^b^	AF^c^
wt TP	100	100	100	100	100	100	137 ± 22
R19A	75 ± 13	102 ± 10	98 ± 27	44 ± 9	93 ± 8	110 ± 8	149 ± 14
K25A	108 ± 17	115 ± 7	87 ± 5	89 ± 3	98 ± 2	121 ± 6	165 ± 27
K27A	21 ± 4	108 ± 7	53 ± 5	3 ± 1	73 ± 9	12 ± 3	18 ± 6
K25A/K27A	19 ± 4	116 ± 9	58 ± 12	3 ± 1	73 ± 5	13 ± 4	19 ± 8
K32A/K33A/K34A	40 ± 17	111 ± 11	99 ± 12	16 ± 5	97 ± 6	104 ± 15	140 ± 9
ΔNt	10 ± 4	109 ± 12	27 ± 4	1 ± 0.3	60 ± 5	3 ± 2	5 ± 3

^a^Numbers indicate the average percentage of activity of the mutant TPs relative to the wild-type TP and the standard deviation obtained from three independent experiments.

^b^% indicates the average percentage of activity of the mutant TPs relative to the wild-type TP and the standard deviation obtained from three independent experiments at 60 min of incubation.

^c^AF indicates the average amplification factor of the mutant TPs relative to the wild-type TP and the standard deviation obtained from three independent experiments at 60 min of incubation.

### The TP N-terminal domain is necessary for a proper interaction with the DNA polymerase

**Figure 3.**
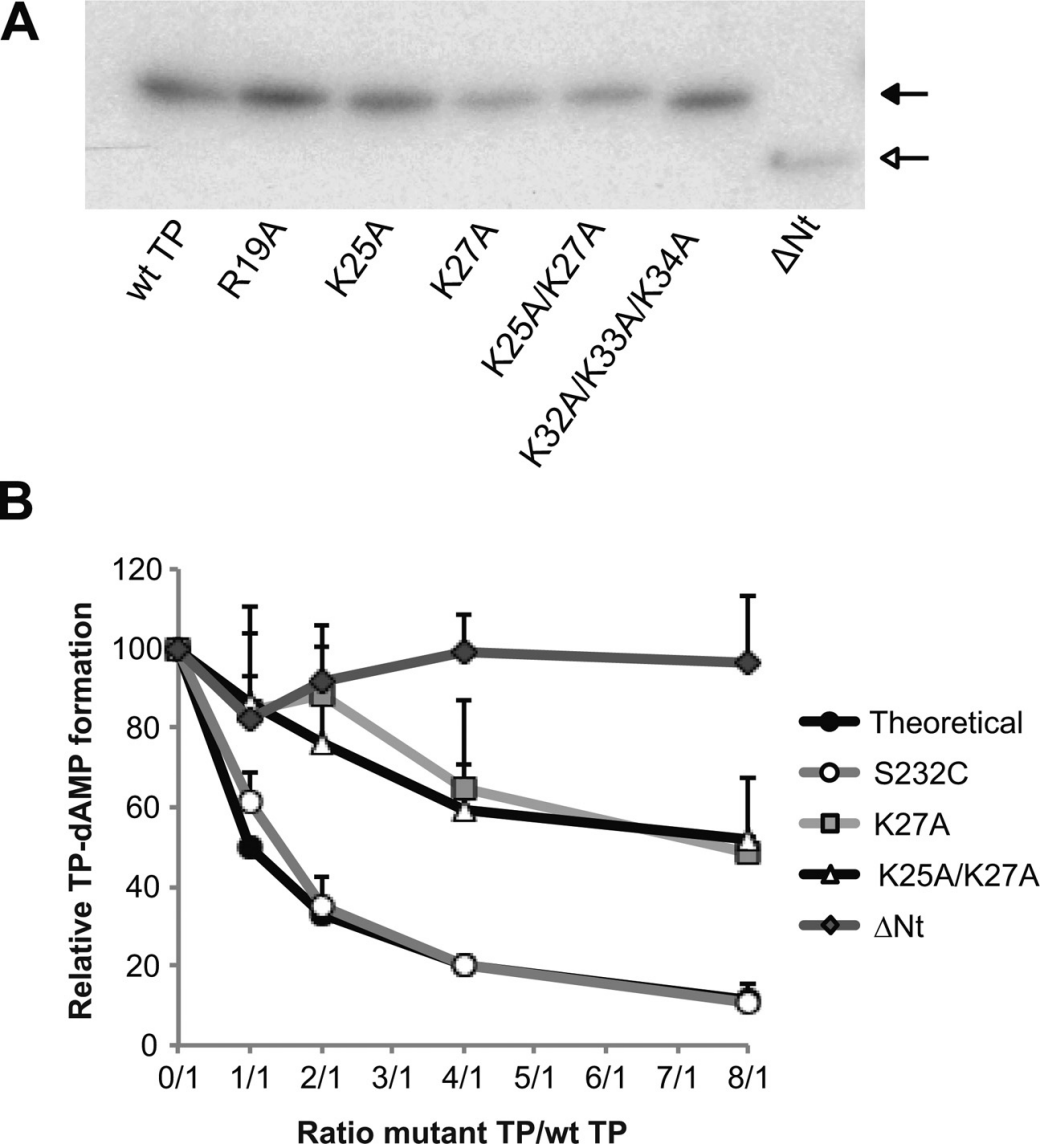
Template-independent TP-deoxynucleotidylation with wild-type and N-terminal mutant TPs. (**A**) TP-dAMP formation with either wild-type or N-terminal mutant TPs in the absence of template. Reactions were carried out as described in Materials and Methods in the presence of 24 nM of TP and 24 nM of DNA polymerase for 1 h at 30°C. Closed arrow indicates the position of TP-dAMP and open arrow indicates the position of ΔNt TP-dAMP. (**B**) Competition for DNA polymerase between wild-type and N-terminal mutant TPs. Reactions were carried out as described for the template-independent TP-dAMP formation assay but adding increasing amounts of mutant TPs. TP-dAMP formation values indicated are relative to those in the absence of competition (100%). As a control, S232C mutant TP was used. Data are represented as mean and standard deviation derived from three independent experiments.

Since the TP mutants affected in performing the TP-dAMP initiation reaction had been shown to have impaired DNA binding capacity (26), we wondered if this initiation impairment was due to a deficiency in the recognition of the replication origin. For this, we made use of the TP-deoxynucleotidylation capacity of ϕ29 DNA polymerase in the absence of template, a reaction strongly dependent on Mn^2+^ ions (36). As shown in Figure 3A and Table 1, TP mutants R19A, K25A and K32A/K33A/K34A showed wild-type efficiencies. In the case of TP mutants K27A and K25A/K27A, they displayed a higher level of initiation activity compared to the wild-type TP than in the template-directed reaction. TP mutant ΔNt also displayed a slightly higher efficiency in these conditions. In the TP-deoxynucleotidylation reaction in the absence of template, the only interactions required are those of the DNA polymerase with the TP and with the initiating nucleotide. Analysis of the interaction between DNA polymerase and TP N-terminal mutants by glycerol gradient sedimentation showed that all mutant TPs formed a stable complex with the DNA polymerase (Supplementary Figure S2). To determine if the impaired formation of the TP ΔNt-dAMP complex in the absence of template was due to a defective interaction with the DNA polymerase, we carried out interference assays in which the interaction of the wild-type TP with the DNA polymerase is competed with increasing amounts of the mutant TP. As a control, we used TP mutant S232C, which has essentially no priming activity but interacts with the DNA polymerase as the wild-type TP (21). As shown in Figure 3B, the inhibition pattern of this mutant TP paralleled the theoretical one. Wild-type TP was competed by mutant TPs K27A and K25A/K27A for the binding to the DNA polymerase, although requiring a higher protein concentration than that of the mutant S232C. On the contrary, the ΔNt mutant TP could not compete with the wild-type TP for interaction with the DNA polymerase, indicating that the interaction of this mutant TP with the DNA polymerase is less efficient than that of the wild-type TP.

### The viral protein p6 overcomes the initiation defect of TP N-terminal mutants affected in DNA binding

Previous work has shown that the ϕ29 dsDNA binding protein p6 stimulates the formation of the TP-dAMP initiation complex by decreasing the K_m_ for the initiating nucleotide (11), as well as the transition from initiation to elongation (12). Protein p6 binds the replication origins and produces a conformational change that was proposed to induce the unwinding of the double helix, hence facilitating the initiation process (13,37,38). To determine if the defect of the TP N-terminal mutants in the initiation of TP-DNA replication was overcome by the action of protein p6, we performed initiation assays using TP-DNA as template as described above, but adding protein p6 to the reaction. Figure 2B and Table 1 show that in the presence of p6 all mutant TPs displayed efficiencies of initiation similar to those of the wild-type TP.

Since binding of the TP mutant ΔNt to the DNA polymerase is less efficient than that of the wild-type TP (see Figure 3B) but its initiation capacity is wild-type-like in the presence of protein p6, we performed template-directed initiation assays either in the absence or in the presence of protein p6 and analysed the capacity of TP mutant ΔNt to compete the wild-type TP for the binding to the DNA polymerase. Supplementary Figure S3 shows that in the absence of protein p6, wild-type TP was only slightly competed by the TP mutant ΔNt. On the contrary, in the presence of protein p6, the inhibition profile of TP mutant ΔNt was similar to that of the TP mutant S232C.

The defined positioning of protein p6 with respect to the DNA ends is required for activation of the initiation reaction (37). For this reason, it had been previously suggested the requirement of protein p6 either for the induction of a conformational change at a specific location in the DNA ends recognised by the heterodimer (38) or for specific interactions with TP and/or DNA polymerase at the origins of replication (38,39). Furthermore, previous results (40) indicated that the N-terminal domain of the ϕ29 DNA polymerase is required for the activation of the initiation reaction by p6, pointing to a possible interaction between p6 and the DNA polymerase. To study these possibilities, we carried out glycerol gradient sedimentation analyses to evaluate the interaction of protein p6 (12 KDa) with either TP (31 KDa) or DNA polymerase (66 KDa). As shown in Supplementary Figure S4A, neither TP nor DNA polymerase formed a stable complex with protein p6 in any of the salt conditions tested. To test the possibility that p6 could interact with TP and/or DNA polymerase but only when the heterodimer has been formed, we carried out glycerol gradient sedimentation analyses with the three proteins present in the reaction simultaneously. Supplementary Figure S4B shows that in these conditions, DNA polymerase and TP interact forming a stable complex, while protein p6 sediments independently in the fractions of lower molecular weight.

### TP N-terminal mutants decrease the affinity of the DNA polymerase for dATP when used as primers and protein p6 restores the wild-type affinity

As mentioned above, protein p6 decreases the K_m_ value of the DNA polymerase for dATP (11). We have shown that TP N-terminal mutants affected in the initiation reaction recovered wild-type-like efficiencies in the presence of protein p6. To test if TP mutants K27A and K25A/K27A affect the affinity of the DNA polymerase for dATP in the initiation reaction, we determined the K_m_ of the DNA polymerase for dATP in TP-DNA templated initiation in the presence of these mutant TPs, both in the absence and in the presence of protein p6. Table 2 shows that in the absence of protein p6, TP mutant K25A gave a K_m_ value similar to that of wild-type TP, whereas TP mutants K27A and K25A/K27A gave rise to a K_m_ value for dATP about 8- and 12-fold higher than the wild-type TP, respectively. As expected, in the presence of protein p6, the K_m_ of the DNA polymerase for dATP was decreased about 9-fold using wild-type TP as primer and 8-fold using K25A TP as primer. Interestingly, using TP mutants K27A and K25A/K27A as primers in the presence of protein p6, the K_m_ of the DNA polymerase for dATP was decreased about 130- and 190-fold, respectively, and was slightly lower (nearly two-fold) than that in the presence of the wild-type TP.

**Table 2.** Nucleotide binding affinity of the DNA polymerase with TP N-terminal mutants either in the absence or in the presence of protein p6

	**K_m_^a^** (μM)	
TP	− p6	+ p6
wt TP	43.5 ± 5.5	4.6 ± 1.5
K25A	33.4 ± 4	4.4 ± 0.7
K27A	354.4 ± 75	2.7 ± 1.4
K25A/K27A	512.3 ± 40	2.7 ± 0.1

**^a^**Michaelis constant values (mean and standard deviation) of the DNA polymerase for dATP in template-directed initiation reactions using the indicated TP as primer, either in the absence or in the presence of protein p6. Values are derived from at least three independent experiments.

### TP N-terminal mutants affected in DNA binding are impaired in the transition from TP-primed initiation to DNA-primed elongation

**Figure 4.**
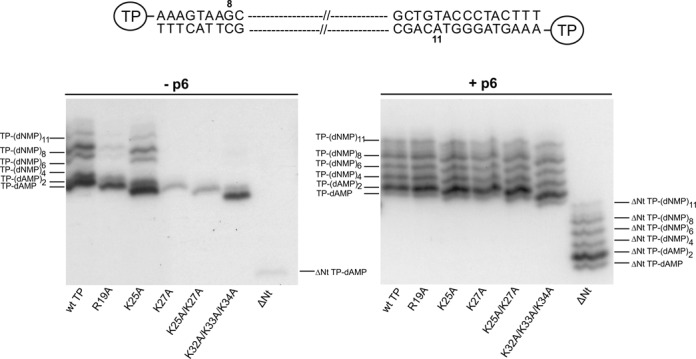
Transition assay with wild-type and N-terminal mutant TPs either in the absence or in the presence of protein p6. Reactions were carried out as described in Materials and Methods. Twelve nM of TP were incubated with 12 nM of DNA polymerase mutant N62D in the presence of 5 μM dATP, dTTP and dGTP (missing dCTP). Samples were incubated at 30°C for 5 min either in the absence or in the presence of 35 μM of protein p6. The position of TP-dAMP initiation and TP-dNMP abortive products are indicated. The figure is representative of three independent experiments. A schematic representation of the two origins of replication and the last products expected from reactions initiating from both ends are depicted on top of the figure.

Once the initiation reaction has taken place, the DNA polymerase remains associated to the TP until the tenth nucleotide is incorporated (8). Then, the DNA polymerase continues processive elongation coupled to strand displacement until completion of replication. The stage between the TP-primed initiation and the DNA-primed elongation is called transition. To analyse the reaction products formed during this stage we used the DNA polymerase mutant N62D, that has low 3′-5′ exonuclease activity (30), to avoid degradation of the replication intermediates. Figure 4 shows that when the wild-type TP is used as primer, the initiation products TP-(dAMP)_1–2_ are generated, as well as TP-(dNMP)_8–11_, corresponding to the truncated elongation initiated from both origins. Besides, a small amount of TP-(dNMP)_4–6_ is produced, probably corresponding to abortive products generated between the initiation and the elongation stage. TP mutant K25A displayed a pattern of products very similar to that of the wild-type TP. In contrast, reaction products with TP mutants R19A and K32A/K33A/K34A were much lower, and only the first band was detected in the case of TP mutants K27A, K25A/K27A, and ΔNt. When protein p6 was added to the reaction, all the mutant TPs presented a pattern similar to that of the wild-type TP. It is worth mentioning that in the presence of protein p6 the product TP-(dAMP)_2_ is accumulated, which could indicate a favoured sliding-back step in these conditions.

### TP N-terminal mutants affected in DNA binding are impaired in ϕ29 TP-DNA replication

**Figure 5.**
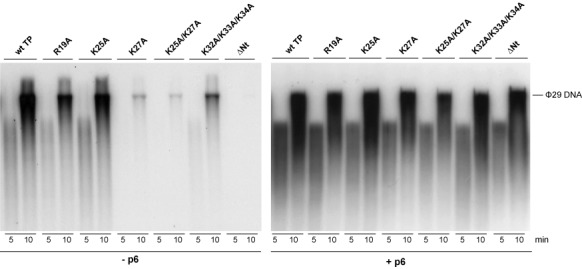
ϕ29 TP-DNA replication with wild-type and N-terminal mutant TPs either in the absence or in the presence of protein p6. Twelve nM of TP were incubated with 12 nM of DNA polymerase for the indicated times in the presence of 20 μM dNTPs. Samples were processed and analysed as described in Materials and Methods. The position of unit length ϕ29 DNA is indicated. The figure is representative of three independent experiments.

Replication of ϕ29 TP-DNA involves protein-primed initiation at both ends of the viral genome and subsequent elongation of the initiation complex by the viral DNA polymerase without the necessity of other proteins, due to its high processivity and strand displacement capacity (18). A minimal replication system *in vitro* has been developed in which only one round of replication is allowed by using TP-DNA as template, and TP and DNA polymerase as the only proteins (18). Figure 5 and Table 1 show that TP N-terminal mutant K25A had a replication efficiency similar to that of the wild-type TP. TP mutant R19A was moderately affected, TP mutant K32A/K33A/K34A was highly affected and TP mutants K27A, K25A/K27A and ΔNt, were severely impaired. Therefore, except K25A TP, all the mutant TPs were affected in a major extent than in the initiation reaction, which could be due to cumulative effects of both impaired initiation and transition. In the presence of the protein p6, the activities of the mutant TPs were similar to those of the wild-type TP, analogous to the effect that protein p6 has on the activity of the mutant TPs for earlier stages in the DNA replication process, i.e. initiation and transition.

### The TP N-terminal domain is essential for an efficient ϕ29 TP-DNA amplification

**Figure 6.**
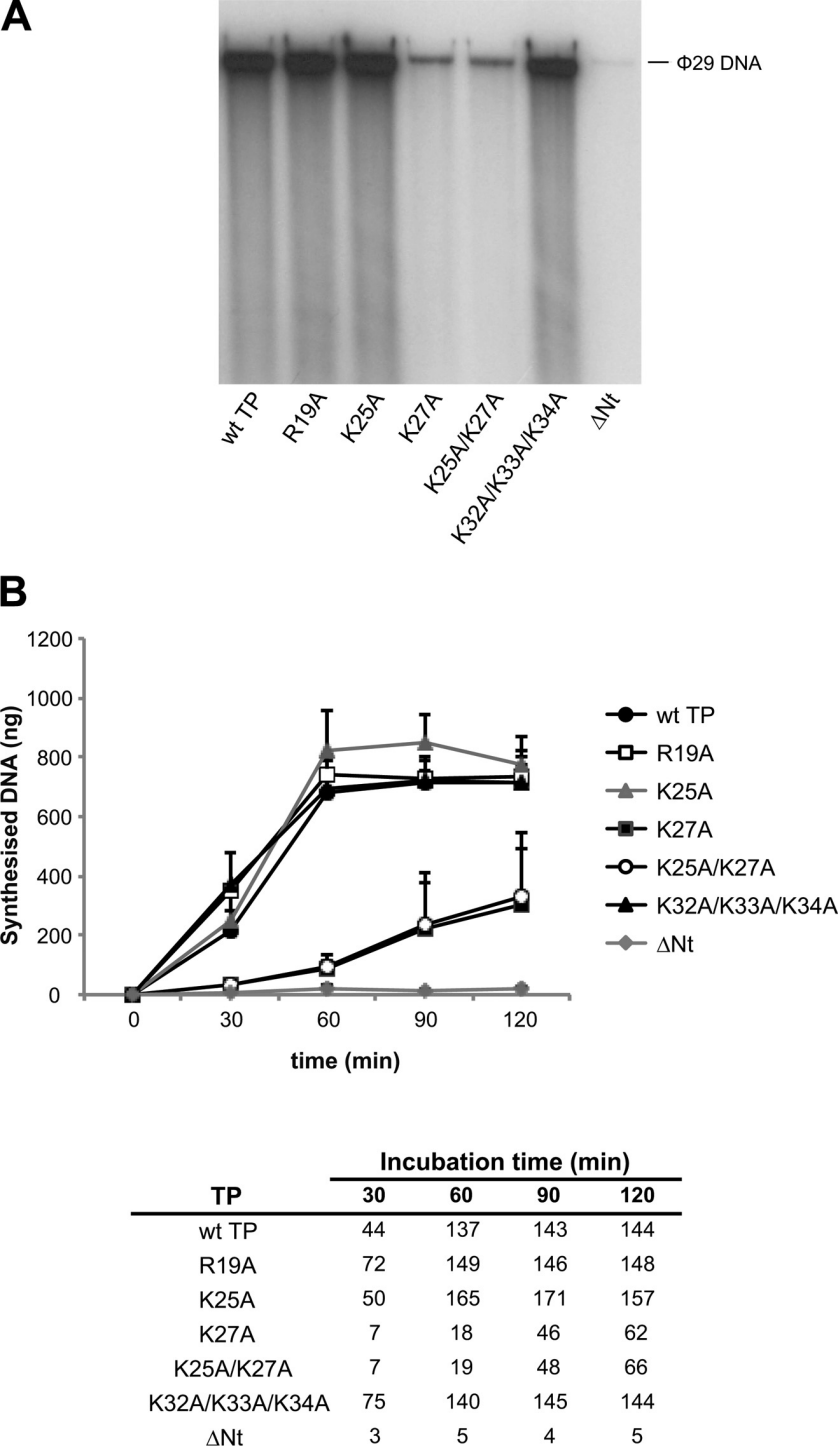
ϕ29 TP-DNA amplification with wild-type and N-terminal mutant TPs. The amplification assay was carried out as described in Materials and Methods with 6 nM of TP and 6 nM of DNA polymerase using 16 pM of TP-DNA as template. (**A**) ϕ29 TP-DNA amplification reactions after 60 min of incubation at 30°C. The position of unit length ϕ29 DNA is indicated. The figure is representative of three independent experiments. (**B**) Kinetics of the synthesised DNA (in ng) at the indicated times of incubation. Data are represented as mean and standard deviation derived from three independent experiments. In the table, numbers show the amplification factors calculated as the ratio between the amount of DNA in ng at the end of the reaction (input DNA plus synthesised DNA) and the amount of input DNA.

After completion of one round of duplication of the viral DNA, the DNA polymerase dissociates to start another round of replication. Limiting amounts (0.5 ng) of input TP-DNA can be amplified up to 1000-fold in the presence of two additional proteins, the ϕ29 SSB protein p5 and the dsDNA binding protein p6 (19). In these conditions, we assayed the amplification capacity of the TP N-terminal mutants. Figure 6A and Table 1 show that TP mutants R19A, K25A and K32A/K33A/K34A displayed wild-type efficiencies. TP mutants K27A and K25A/K27A gave values of 12–13% of the wild-type TP at the time when the reaction with the wild-type protein had gone to completion (60 min). Figure 6B presents the kinetics of the synthesised DNA and the amplification factor obtained at the different times post-incubation with each TP. The amplification factor describes the ratio between the amount of DNA in ng at the end of the reaction (input DNA plus synthesised DNA) and the amount of input DNA. At 60 min post-incubation, the amplification factor when the wild-type TP is used as primer is 137, while the amplification factor with TP mutants K27A and K25A/K27A is 18 and 19, respectively. However, at longer incubation times, these proteins continued synthesising viral DNA; their amplification factors after 120 min were 62 and 66, respectively. On the contrary, TP mutant ΔNt was stalled at amplification factors of 4–5 even at the longest incubation time (120 min).

**Figure 7.**
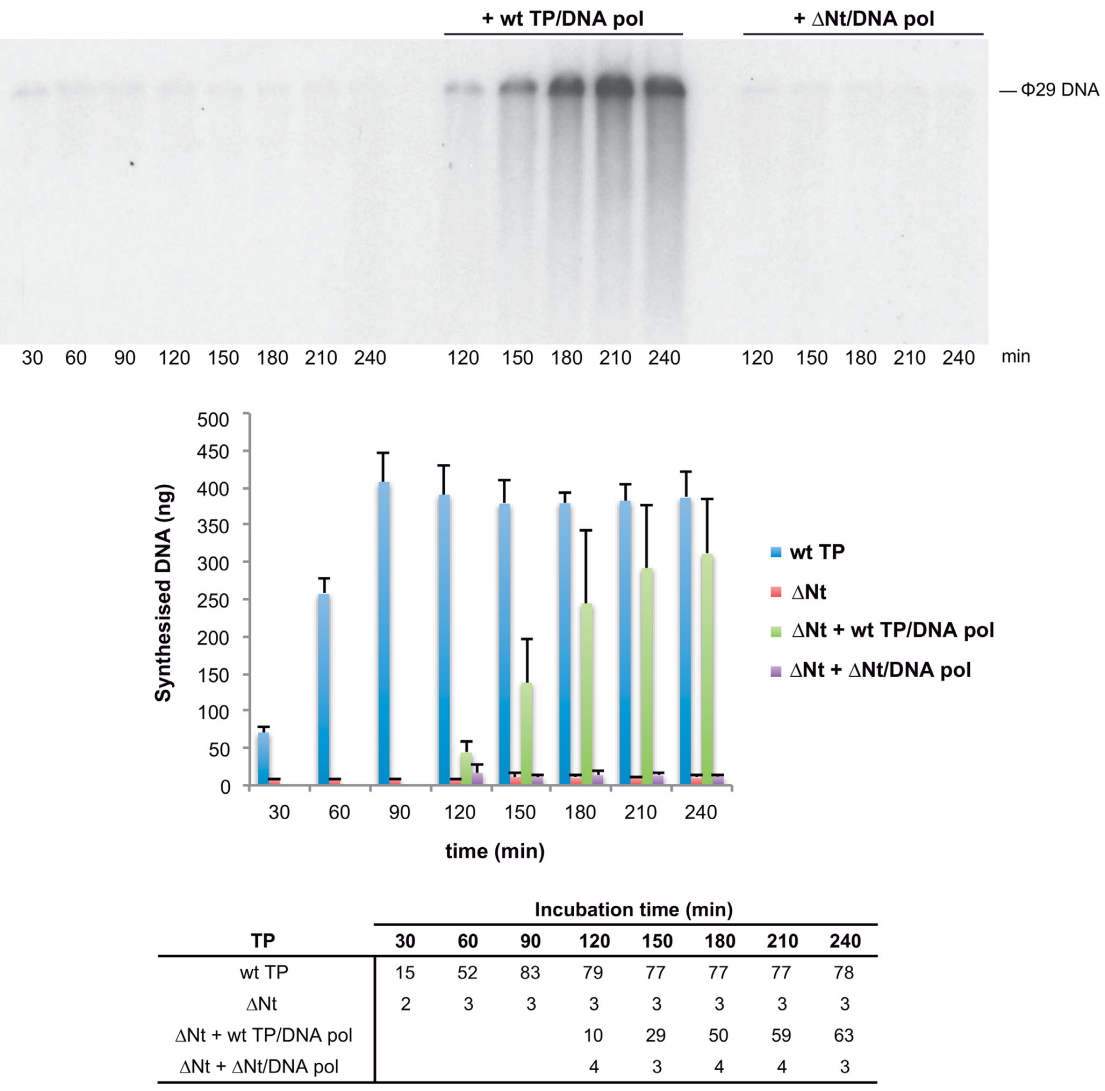
Amplification with TP mutant ΔNt is recovered upon addition of wild-type TP/DNA polymerase heterodimer but not upon addition of TP ΔNt/DNA polymerase heterodimer. The upper panel shows the amplification reactions with TP mutant ΔNt at the indicated times and the amplification reactions after the addition of either wild-type TP/DNA polymerase heterodimer or TP ΔNt/DNA polymerase heterodimer at 90 min post-incubation. The position of unit length ϕ29 DNA is indicated. The figure is representative of three independent experiments. The bar graph shows the kinetics of DNA synthesis in ng for each of the above-mentioned conditions at the indicated times. Bars represent mean and standard deviation derived from three independent experiments. The bottom panel shows the amplification factors calculated as the ratio between the amount of DNA in ng at the end of the reaction (input DNA plus synthesised DNA) and the amount of input DNA.

In amplification reactions the TP acts first as a primer, but in the next replication round the same TP molecule becomes parental TP. To assess if the defect on TP-DNA amplification of the TP mutant ΔNt was due to an impairment in its capacity as parental TP, and thus to inactivation of the origins of replication for further initiation events, we performed amplification assays with this mutant TP for 90 min, and then added to the reaction either wild-type TP/DNA polymerase heterodimer or mutant TP ΔNt/DNA polymerase heterodimer. As shown in Figure 7, amplification only proceeded when the wild-type heterodimer was added. Although mutant TP ΔNt can serve as parental TP in the presence of wild-type primer TP, the origins of replication are impaired for initiation when mutant TP ΔNt is present simultaneously as parental and primer TP. This impairment could be due to a defective interaction between primer and parental ΔNt TPs during the initiation reaction. In a similar manner, since amplification reactions were less efficient with TP mutants K27A and K25A/K27A, these mutant TPs could be moderately affected in serving as parental TP.

## DISCUSSION

The correct achievement of the first steps of DNA replication, in which multiple and specific protein–protein and DNA–protein interactions occur, is crucial for the efficiency of this process. Initiation of ϕ29 DNA replication takes place by the so-called protein-priming mechanism. Both the initiation reaction and the transition from initiation to the elongation stage have been shown to be stimulated by the viral protein p6, which lowers the K_m_ of the DNA polymerase for the initiating nucleotide (10–12). Here we show that TP N-terminal mutants affected in DNA binding are impaired in the initiation and further elongation of ϕ29 TP-DNA replication, and that protein p6 is crucial to relieve these defects. Accordingly, using TP N-terminal mutants K27A, K25A/K27A and ΔNt as primers, only a small amount of the initiation product TP-dAMP is formed, and the reaction is blocked for further incorporation events unless protein p6 is provided (Figure 4). It has been described that the initiation reaction requires a lower dATP concentration than the incorporation of the second and further residues (11,12). These data might suggest that the improvement of the initiation and transition reactions with the TP N-terminal mutants when p6 is present is due to a decrease in the K_m_ of the DNA polymerase for dATP. Measurement of the K_m_ for dATP in the absence of protein p6 using either K27A or K25A/K27A mutant TPs as primers showed that the DNA polymerase has a K_m_ for dATP about 8 and 12 times higher, respectively, than when wild-type TP is used. In contrast, in the presence of protein p6, the DNA polymerase has low K_m_ values for dATP using either the wild-type or the mutant TPs. In fact, the K_m_ for dATP of the DNA polymerase when TP N-terminal mutants K27A and K25A/K27A are used as primers in the presence of protein p6 is nearly 2-fold lower than when wild-type TP is used. This could be due to a more favourable positioning of the dATP at the active site of the DNA polymerase with these mutant TPs with respect to the positioning displayed with the wild-type TP.

In the absence of protein p6, the increase of the K_m_ value for dATP when these mutant TPs are used as primers could be due to a defective positioning of the hydroxyl group of Ser232 in the DNA polymerase active site. It is likely that binding of the TP to the template DNA contributes to the proper positioning of Ser232 in the active site of the DNA polymerase. Accordingly, when TP N-terminal mutants affected in DNA binding are used as primers in initiation reactions in the absence of template, their efficiencies are closer to those of the wild-type TP (Figure 3A). Furthermore, the defective positioning in TP N-terminal mutants affected in DNA binding may be relieved by the presence of protein p6, which has been described to change the conformation of DNA upon binding (13), and thereby could favour the binding of TP.

The requirement of a precise positioning of p6 at the origin of replication with respect to the DNA ends to activate initiation (37) might suggest a direct interaction between p6 and either the DNA polymerase and/or the TP. Additionally, analysis of the ability of the p6 proteins from ϕ29 and the related phages Nf and GA1 to activate the initiation reaction showed that optimal activation required the formation of the homologous nucleoprotein complex at the origins, i.e. p6 and TP-DNA coming from the same phage, as well as the recognition of this complex by the homologous TP/DNA polymerase heterodimer (39), further supporting the hypothesis of a specific interaction between p6 and either the DNA polymerase and/or the TP. In the present work, we could not detect the formation of a stable complex between p6 and either the DNA polymerase or the TP by glycerol gradient sedimentation. However, this putative interaction might be transient or DNA dependent, and therefore might not be detected by this method. It is important to note that protein p6 forms a dynamic complex with DNA (41) and a tight interaction between the DNA polymerase and protein p6 would be unfavourable for a rapid amplification of the template DNA.

By interference assays we have shown that deletion of the TP N-terminal domain affects its interaction with the DNA polymerase. Although not making direct contacts with the DNA polymerase (9), the N-terminal domain of the TP could be involved in providing the entire TP structure with a proper conformation to interact with the DNA polymerase. Indeed, it has been shown that a deletion mutant of the TP comprising only the C-terminal domain (priming domain) cannot perform the initiation reaction unless the intermediate plus N-terminal domain is provided in *trans*, which was proposed to induce a conformational change in the DNA polymerase that would allow the priming domain to be correctly positioned at the polymerase catalytic site (20). As shown above, TP mutant ΔNt performed the initiation reaction efficiently only in the presence of protein p6; indeed, it can only compete with wild-type TP for the binding with the DNA polymerase in the template-directed reactions in the presence of protein p6, which suggests that p6 promotes a proper interaction between TP mutant ΔNt and the DNA polymerase.

As described in this work, the TP N-terminal domain is essential for ϕ29 TP-DNA amplification *in vitro*, since the reactions with the TP mutant ΔNt were stalled at amplification factors of 4–5. Once one round of replication was achieved and TP mutant ΔNt became parental TP, further replication of the mutant TP-containing origin was not allowed, unless provided with wild-type TP/DNA polymerase heterodimer. TP mutants affected in ϕ29 TP-DNA amplification that were recovered upon addition of wild-type TP/DNA polymerase heterodimer have been reported (23,24). The observation that TP molecules can interact with each other (42–45) further suggests that recruitment of the heterodimer to the origin is achieved by protein–protein contacts between the parental and primer TP. Therefore, it seems likely that the deletion of the TP N-terminal domain affects directly TP/TP contacts, precluding in this way the primer TP/parental TP interaction. Together, these results suggest that impairment of the origins of replication is caused by the lack of functional interactions between primer ΔNt and parental ΔNt TPs. This could be explained by a model in which the N-terminal domain of one TP would interact with the intermediate and/or the C-terminal domain of the other TP involved in the interaction. The fact that the amplification reactions were recovered upon addition of wild-type TP heterodimers points out to the necessity of a complete N-terminal domain at least in one of the TPs involved in the interaction.

In *B. subtilis* infected cells, protein p6 is present at high amounts (about 700 000 molecules per cell at late post-infection times) (46). As it was suggested for the adenovirus DNA binding protein DBP (47), the K_m_-reducing activity of p6 could compensate for an eventual low availability of dNTPs to the viral replication machinery, facilitating the efficiency of the phage DNA replication *in vivo*. In line with the results presented in this paper, it has been shown that mutations in adenovirus pTP (precursor terminal protein) residues Asp578 and Asp582, close to the priming residue Ser580, affected the kinetics of initiation by increasing the K_m_ of the DNA polymerase for dCMP incorporation (48). Furthermore, it was shown that pTP mutants affected in DNA binding were impaired in the initiation of adenovirus replication, suggesting that the pTP binding is important for this process (49). It has been proposed that the DNA binding capacity of the pTP and its potential interaction with the parental TP could be stabilising the pTP/Ad pol heterodimer at the origins of replication (50).

In the case of ϕ29, up to now only mutants in DNA polymerase residues had been shown to affect the kinetics of initiation (51–55). The results presented in this paper show the involvement of ϕ29 TP N-terminal domain DNA binding residues in the kinetics of initiation of viral DNA replication and stresses the importance of the TP N-terminal domain both for a proper interaction with the DNA polymerase and for TP-DNA amplification *in vitro*. We propose a model in which the binding of the TP to the template DNA allows the proper positioning of the TP initiating residue at the active site of the DNA polymerase. It is important to note that TP N-terminal residues involved in DNA binding are highly conserved in the ϕ29-like bacteriophages PZA, Nf, B103 and GA1 (26), suggesting an essential role of these residues for viral DNA replication. Furthermore, we provide further insights into the contribution of protein p6 to the efficient achievement of the first stages of ϕ29 TP-DNA replication: protein p6 reduces the K_m_ of the DNA polymerase for the initiating nucleotide when TP mutants affected in DNA binding are used as primers. Based on our results, we pose the hypothesis that protein p6 allows the proper positioning of the TP at the active site of the DNA polymerase by promoting a conformational change in the template DNA that is recognised by the TP.

## SUPPLEMENTARY DATA

Supplementary Data are available at NAR Online.

SUPPLEMENTARY DATA
