# Supplementary material for: Dissecting the role of the ϕ29 terminal protein DNA binding residues in viral DNA replication
Source: Nucleic Acids Res. 2015 Feb 26;43(5):2790–801. doi: 10.1093/nar/gkv127 (PMC4357725; doi:10.1093/nar/gkv127)
Supplement: SUPPLEMENTARY DATA [file supp_43_5_2790__index.html]

Dissecting the role of the ϕ29 terminal protein DNA binding residues in viral DNA replication — SUPPLEMENTARY DATA 

# Dissecting the role of the ϕ29 terminal protein DNA binding residues in viral DNA replication

## SUPPLEMENTARY DATA

**Files in this Data Supplement:**

- SUPPLEMENTARY DATA
