# Supplementary material for: Dissecting the role of the ϕ29 terminal protein DNA binding residues in viral DNA replication
Source: Nucleic Acids Res. 2015 Feb 26;43(5):2790–801. doi: 10.1093/nar/gkv127 (PMC4357725; doi:10.1093/nar/gkv127)
Supplement: SUPPLEMENTARY DATA [file supp_gkv127_nar-03267-m-2014-File009.pdf]

SUPPLEMENTARY DATA

SUPPLEMENTARY METHODS

Analysis of the interaction of N-terminal mutant TPs with DNA polymerase by glycerol gradient sedimentation

The incubation mixture contained, in 200 µl, 50 mM Tris-HCl, pH 7.5, 0.1 mg/ml BSA, 20 mM ammonium sulphate, 1 mM DTT, 448 nM of either wild-type or N-terminal mutant TP and 448 nM of DNA polymerase. Samples were incubated for 30 minutes at 4 ºC, then loaded on top of a continuous 15%–30% (v/v) glycerol gradient (4 ml) in the presence of 50 mM Tris–HCl, pH 7.5, 20 mM ammonium sulphate, 180 mM NaCl, 1 mM EDTA and 7 mM 2-mercaptoethanol, and centrifuged at 4 ºC for 24 hours at 58,000 rpm in a Beckman TST 60.4 rotor. Gradients were fractionated and subjected to SDS-12% PAGE. Gels were stained after electrophoresis with SYPRO® Ruby to identify the peaks corresponding to the TP/DNA polymerase heterodimer (97 kDa) and the free monomers of DNA polymerase (66 kDa) and TP (31 kDa).

Analysis of the interaction of wild-type TP and DNA polymerase with protein p6 by glycerol gradient sedimentation

The incubation mixture contained, in 200 µl, 50 mM Tris-HCl, pH 7.5, 0.1 mg/ml BSA, 20 mM ammonium sulphate, 1 mM DTT, 713 nM of p6 and either 713 nM of wild-type TP or 713 nM of DNA polymerase. In the case of the incubation of the three proteins simultaneously, the incubation mixture contained, in 200 µl, 50 mM Tris-HCl, pH 7.5, 0.1 mg/ml BSA, 20 mM ammonium sulphate, 1 mM DTT, 713 nM of wild-type TP, 713 nM of DNA polymerase and 713 nM of p6. Gradients (prepared as described above with either 90 or 180 mM NaCl) were fractionated and subjected to SDS-10-20% PAGE. Gels were stained after electrophoresis with SYPRO® Ruby to identify the peaks corresponding to the TP/DNA polymerase heterodimer (97 kDa), DNA polymerase (66 kDa), TP (31 kDa) and p6 (12 kDa).

SUPPLEMENTARY REFERENCE

1. de Vega, M. and Salas, M. (2011) In Kusic-Tima, J. (ed.), *DNA replication and related cellular processes*, pp. 179-206.

SUPPLEMENTARY FIGURES

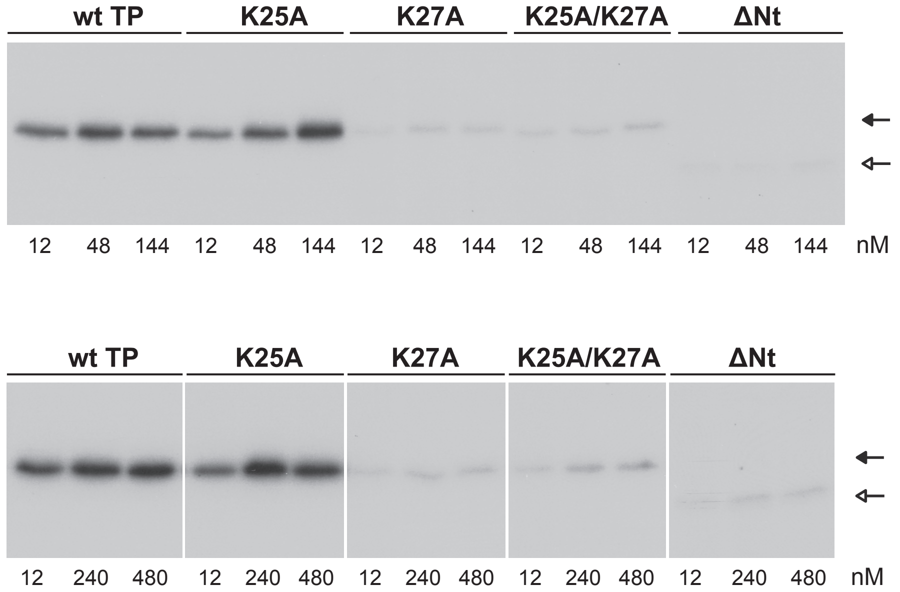

Supplementary Figure S1

Protein-primed initiation of φ29 TP-DNA replication with TP N-terminal mutants. The indicated amounts of either wild-type or N-terminal mutant TPs were incubated with 12 nM DNA polymerase for 4 min at 30 ºC, using 1.6 nM of TP-DNA as template and 10 mM $MgCl_2$ as metal activator. The samples were processed as described in Materials and Methods for the protein-primed initiation assay.

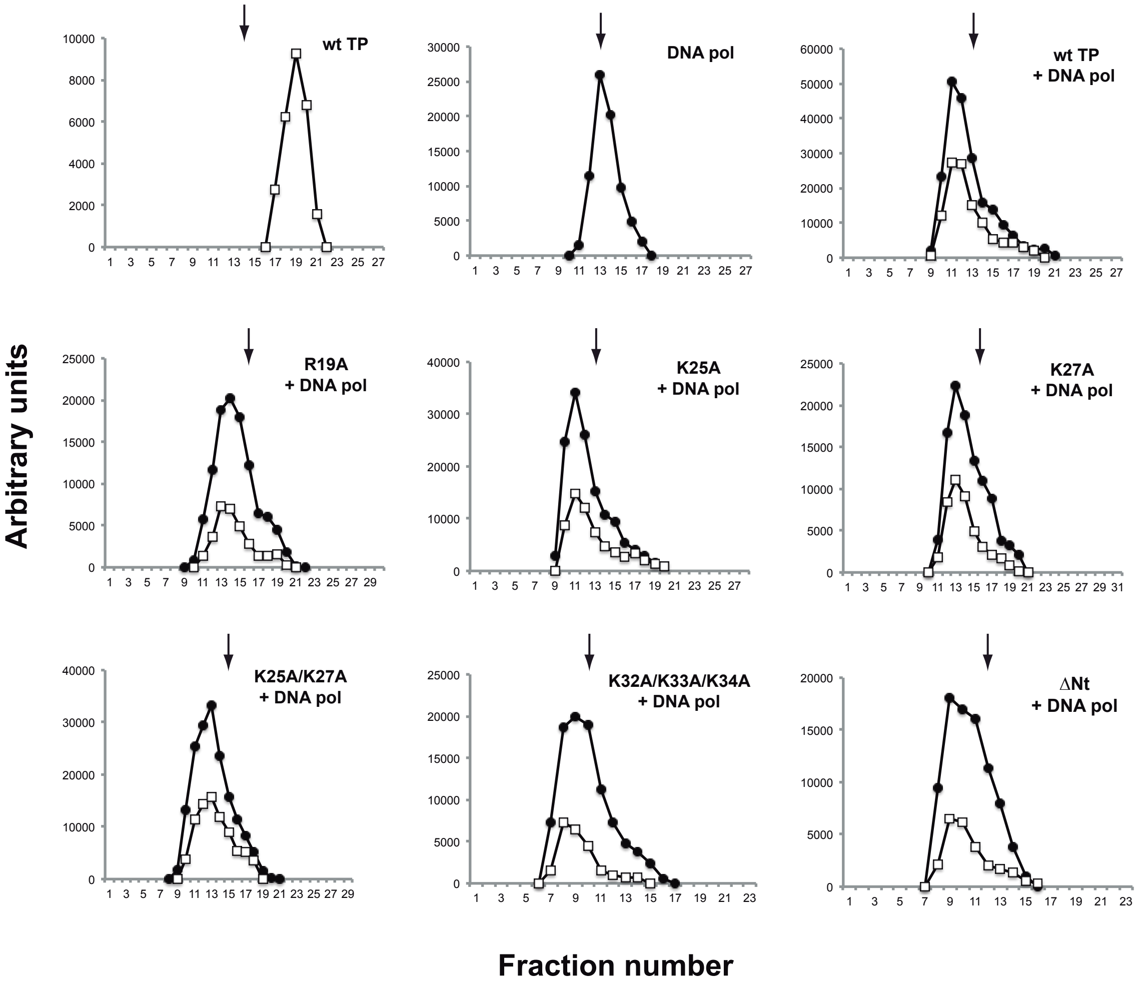

Supplementary Figure S2

Analysis of N-terminal mutant TPs/DNA polymerase interaction by glycerol gradient sedimentation. 448 nM of either wild-type or N-terminal mutant TPs were pre-incubated with 448 nM of DNA polymerase for 30 min at 4 ºC and loaded on top of a continuous 15-30% (v/v) glycerol gradient in the presence of 180 mM NaCl. Gradients were centrifuged and processed as described in Materials and Methods. Densitometric quantification, expressed in arbitrary units, of DNA polymerase (black circles) and either wild-type or N-terminal mutant TPs (white squares) is represented. An arrow denotes the BSA peak. Fractions are numbered from bottom to top.

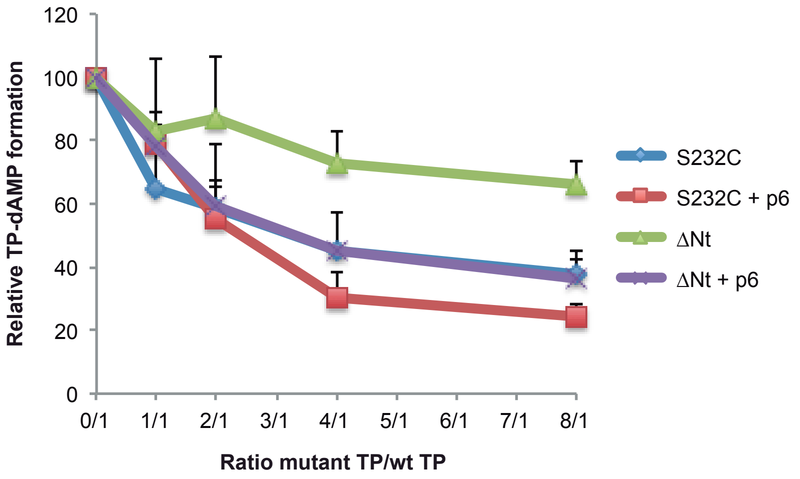

Supplementary Figure S3

Competition for DNA polymerase binding between wild-type TP and TP mutant ΔNt either in the absence or in the presence of protein p6. Reactions were carried out as described for the template-directed initiation assay, but adding increasing amounts of TP mutant ΔNt. TP-dAMP formation values indicated are relative to those in the absence of competition (100%). As a control, S232C mutant TP was assayed either in the absence or in the presence of protein p6. Data are represented as mean and standard deviation derived from three independent experiments.

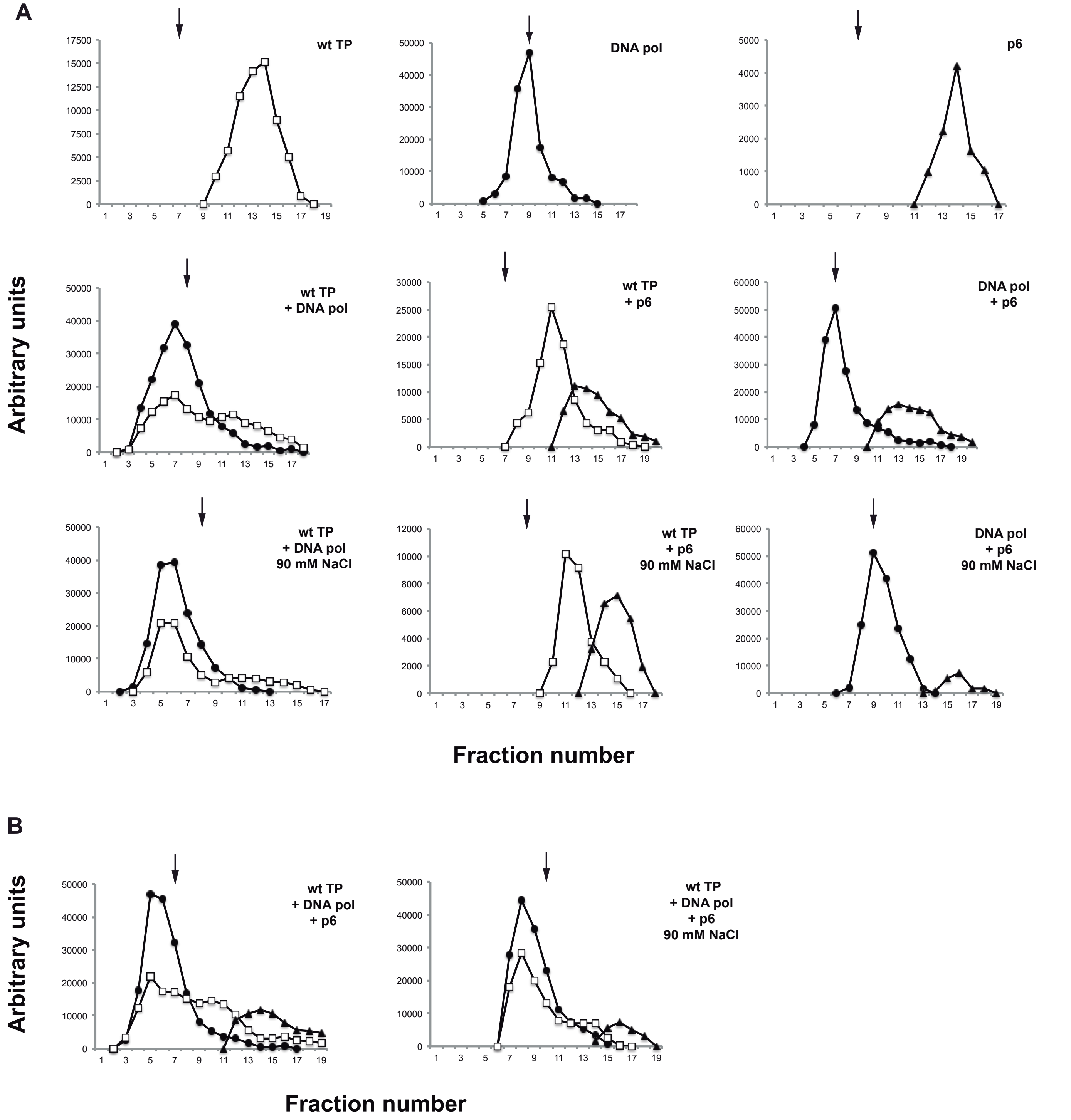

Supplementary Figure S4

(A) Analysis of the interaction of protein p6 with either wild-type TP or DNA polymerase by glycerol gradient sedimentation. 713 nM of p6 were pre-incubated with either 713 nM of wild-type TP or 713 nM of DNA polymerase for 30 min at 4 ºC and loaded on top of a continuous 15-30% (v/v) glycerol gradient in the presence of 180 mM NaCl except where indicated. Gradients were centrifuged and processed as described in Materials and Methods. Densitometric quantification, expressed in arbitrary units, of DNA polymerase (black circles), wild-type TP (white squares), and p6 (black triangles) is represented. An arrow denotes the BSA peak. Fractions are numbered from bottom to top. **(B)** Analysis of the interaction of protein p6 with either wild-type TP or DNA polymerase in the presence of both proteins simultaneously. 713 nM of p6 were pre-incubated with 713 nM of wild-type TP and 713 nM of DNA polymerase for 30 min at 4 ºC and processed as described above. Symbols are as in A.
